# 肿瘤患者对临床试验认知度及其影响因素的调查分析

**DOI:** 10.3779/j.issn.1009-3419.2020.01.02

**Published:** 2020-01-20

**Authors:** 慧瑶 黄, 元 方, 虹 房, 大维 吴, 颖 白, 书航 王, 安琪 于, 辉 王, 超 孙, 琦 樊, 悦 俞, 晟 杨, 菊芳 石, 瑞仙 何, 宁 李

**Affiliations:** 100021 北京，国家癌症中心/国家肿瘤临床医学研究中心/中国医学科学院北京协和医学院肿瘤医院 Department of Clinical Trials Center, National Cancer Center/National Clinical Research Center for Cancer/Cancer Hospital, Chinese Academy of Medical Sciences and Peking Union Medical College, Beijing 100021, China

**Keywords:** 临床试验, 认知度, 知情同意, 癌症, Clinical trial, Awareness, Informed consent, Cancer

## Abstract

**背景与目的:**

早期研究表明患者对临床试验的认知度是影响其参与度的重要因素。本文主要为掌握中国肿瘤患者临床试验的认知度情况并探索相关影响因素，比较参加过和未参加试验患者认知度差异。

**方法:**

2018年6月-2019年4月，采用标准化问卷收集中国医学科学院肿瘤医院肿瘤患者（参加过*vs*未参加过试验）基本信息、对临床试验整体看法及其他认知相关的10个维度，计算认知度合计得分，重新分为“认知较高组”和“认知较低组”，采用二元*Logistics*进行认知度的单因素和多因素影响分析。

**结果:**

共纳入617例肿瘤患者，38.6%患者参加过试验。338例（54.6%）对试验整体看法认知正确，但仍有44例（7.1%）患者认为“参与临床试验患者是科学研究的牺牲品”。除外试验补偿维度（51.5% *vs* 48.7%）和法律法规（52.3% *vs* 45.5%）维度，参加过试验患者在研究意义（86.2% *vs* 77.6%）、风险收益告知（91.2% *vs* 71.6%）、资料保密（73.2% *vs* 59.7%）、自愿参与（95.8% *vs* 76.3%）、随时退出（86.6% *vs* 68.2%）、费用影响（62.8% *vs* 39.2%）6个维度相比未参加过试验患者认知正确比例均有一定提高。多因素分析结果显示，参加过试验（OR=1.83, 95%CI: 1.11-3.00）、未婚/离异（OR=5.04, 95%CI: 1.73-14.66）、退休（OR=2.53, 95%CI: 1.16-5.50）患者认知度较高，对医务人员印象一般/差（OR=0.43, 95%CI: 0.26-0.72）者认知度较低。

**结论:**

我国肿瘤患者对临床试验的认知度较为有限，包括参加过试验患者。促进医患和谐、开展临床试验知识普及对提高患者认知度十分有必要；同时，针对性地加强临床试验知情同意告知的充分性和有效性也是未来工作的重要方向。

## Abstract (en)

**Background and objective:**

Early investigation suggested patients' level of awareness regarding clinical trials was related with willingness to participation. This study was intended to evaluate the level of awareness of cancer patients regarding clinical trials and related influencing factors, and to compare the differences of awareness between patients who attended clinical trials before and not.

**Methods:**

From Jun, 2018 to April, 2019, standardized question-naires were gathered from cancer patients (attended clinical trials *vs* not attended clinical trials) in our hospital regarding basic information and 10 other questions about awareness. The level of awareness was evaluated and patients were classified into "low cognition" and "high cognition" groups. *Logistic* regression analysis was performed to determine whether certain characteristics would predict for awareness.

**Results:**

Of the 617 participants, 38.6% have attended clinical trials before. 338 (54.6%) patients had a correct overall understanding of clinical trials, while 44 (7.1%) patients still thought participants were the victim of scientific research. Except for the compensation of medical expenses (51.5% *vs* 48.7%) and related laws of clinical trials (52.3% *vs* 45.5%), other parts of understanding were elevated in patients attended clinical trials before comparing with patients who didn't, including significance (86.2% *vs* 77.6%), risk disclosure (91.2% *vs* 71.6%), confidentiality (73.2% *vs* 59.7%), voluntariness (95.8% *vs* 76.3%), withdrawal (86.6% *vs* 68.2%) and expenses (62.8% *vs* 39.2%). The proportion of participants who understand these components did not increase even in 239 patients who had attended clinical trials before. Participants who attended clinical trials before (OR=1.83, 95%CI: 1.11-3.00), unmarried/divorced (OR=5.04, 95%CI: 1.73-14.66), retired (OR=2.53, 95%CI: 1.16-5.50) had a higher level of awareness, while patients who had bad impression with doctors (OR=0.43, 95%CI: 0.26-0.72) had lower awareness.

**Conclusion:**

The current level of awareness for clinical trials of cancer patients in our hospital was relatively low, even in patients who had attended clinical trials before. It's necessary to improve patients' awareness of clinical trial by promoting harmony relationship between patients and doctors, as well as by enhancing related propagation. Strengthening the adequacy and efficacy of informed consent in clinical trials also needs to be achieved in the future.

近年来，越来越多的抗肿瘤药物临床试验在我国开展^[1]^。2018年，中国新增注册肿瘤药物临床试验322项，参与试验总人数达49, 517，是2017年的3.2倍^[2]^。然而，相对于我国每年新发肿瘤患者，参加临床试验的患者仍然有限^[3]^。患者招募困难和入组速度受限仍是现阶段开展临床试验的一大阻碍，增加研发成本，同时延长了有效药物获批惠及百姓的时间^[4, 5]^。

早期研究^[6, 7]^表明患者对于临床试验的认知度是影响其参与度的重要因素。近几年，越来越多的国外医疗机构认识到提高民众对临床试验认知度的重要性。美国国家卫生研究院设立了针对临床试验的科普网站，并举行了大型的科教活动^[8, 9]^。这些举措使美国公众对于临床研究的知晓率在4年间从68%上升至74%^[10]^。然而，相关的科普宣传在我国很少开展，缺乏对患者认知状态的全面评估是其中的重要原因之一。我国少量单中心研究数据表明超过半数肿瘤患者没有正确认识临床试验^[11, 12]^，但关于认知欠缺的具体方面以及影响认知度的因素仍知之甚少；同时，针对临床试验受试者的知情同意是否充分也缺乏相关研究证据。

因此，本研究基于国家癌症中心/中国医学科学院肿瘤医院，采用标准化问卷调查参加过试验和未参加过试验的肿瘤患者，评估两组患者对于试验的认知情况并探索相关影响因素，为今后我国开展有针对性的的临床试验科普宣传活动提供数据支持，以提高患者临床试验认知度和参与度。

## 1 资料与方法

### 1.1 研究对象

从2018年4月-2018年12月，针对正在或曾在中国医学科学院肿瘤医院诊疗或曾参加临床试验的恶性肿瘤患者及其家属开展问卷调查，采用分层方便抽样，控制门诊和住院比例为1:1，内科、外科、放疗比例为2:1:1。研究的纳入标准包括：①明确诊断的恶性肿瘤患者或家属；②年龄≥18岁；③意识清楚，听力正常，无精神心理疾病；排除诊断不明确或者良性肿瘤患者及家属。本研究选取了肿瘤患者，包括：①参加过试验的患者（既往参加过或正在参加）；②未参加过试验的患者作为研究对象，所有调查对象均签署了知情同意书。研究通过了中国医学科学院肿瘤医院伦理委员会的审查（批准批号：18-028/1629）。

### 1.2 研究方法

本研究基于统一编制的问卷开展调查。所有被调查对象均需进行参与情况登记，包括基本信息（性别、年龄、对象来源）、是否参加过临床试验、是否参与本次调查等。若拒绝调查，记录原因；若参加调查，分配ID号，并在签署知情同意书后发放调查主表“肿瘤患者及家属对临床试验研究的认知和接受度调查”。

调查主表主要包括以下5个部分。①社会人口学信息：包括年龄、性别、婚姻状况、职业、教育程度、宗教、对于健康节目关注度、有无医疗行业亲友、对于医务人员整体印象等。②临床信息：包括患者发病时间、肿瘤分期、癌变部位、治疗方案、治疗阶段、自评疗效、医疗费用、医保类型及自评经济压力等。③临床试验的认知情况：包括整体看法、试验意义、试验目的、风险和受益告知、资料保密、自愿参与、随时退出、医疗费用影响、损害补偿和法律法规。④临床试验选择倾向和接受度：包括是否参加过临床试验、是否愿意参加或推荐亲友参加、原因和不愿意的主要原因、对试验了解的需求、希望获取的试验信息内容和渠道、参加过临床试验的期别、参加的主要原因等。⑤调查员后记：调查对象合作情况、对上述4个板块信息可靠性评价、调查员和审核员签字和日期。

### 1.3 质量控制

为保证本调查的研究质量，研究团队主要从问卷开发、人员培训及多级质控等3个方面进行质量控制。①问卷开发：问卷设计通过国家癌症研究中心专家研讨确认其科学性；同时，通过开展预调查（30例）进行问卷修改和完善。②人员培训：问卷调查员均为驻点中国医学科学院肿瘤医院的临床协调员，研究团队对调查人员进行资质审核和统一培训，培训内容包括研究方案、调查内容、调查标准化流程、沟通技巧及注意事项等。③多级质控：除外问卷第五部分设计调查员后记，以评估调查对象合作情况和数据可靠性情况外，问卷调查完成后要求调查员自检，2 d内质控员完成质控复检，数据统一上报后由研究数据管理员采用SAS软件再次核查，对于缺失/错误数据进行补充修改，若关键变量仍缺失/错误，则在数据分析阶段剔除记录。

### 1.4 统计处理及分析

调查数据录入Epidata 3.1软件，采用SAS 9.4对数据分析和管理。满足以下任何一条的即予以剔除：①年龄缺失；②性别缺失；③是否参加过试验；④临床试验整体看法缺失。结果变量除外9个具体认知问题回答正确人群的比例，本研究依据以下规则将总体认知度进行二分类（“认知较低”和“认知较高”），归类过程如下：①每题认知正确者得1分，其他为0分；②计算认知得分合计，范围为0分-9分；③将纳入对象认知得分合计排序，取中位数作为界值，高于界值者归为“认知较高”组，否则归为“认知较低”组。

除外年龄和确诊时间为计量资料，统计描述采用“均数±标准差”，其余均为计数资料，采用频数（%）表示；年龄和确诊时间均不服从正态分布，统计分析采用秩和检验，其他计数资料采用卡方检验。针对总体认知度，纳入14个解释变量先进行单因素分析，再以逐步回归法进行二元*Logistic*模型进行多因素影响分析，进入标准为*P* < 0.10，剔除标准为*P* > 0.15。所有统计检验以*P* < 0.05为差异有统计学意义。

## 2 结果

### 2.1 基本信息

研究向696例患者发放调查问卷，回收问卷625份，研究参与率89.8%。最终纳入分析619例患者数据进行统计分析。调查员评价对象合作情况显示，有70.8%和21.0%患者合作很好和较好。

详细的人口学信息见表 1。38.6%的患者参加过临床试验，61.4%的患者未参加过临床试验患者，平均年龄为53.4岁，大部分文化水平是高中/中专/大专及以下（80.2%），职业方面占比最高为公司职员、公务员、事业单位人员（34.3%）。参加过临床试验和未参加临床试验的肿瘤患者在年龄、性别、婚姻、文化程度、职业、宗教信仰上的分布无统计学差异（表 1），但参加过临床试验的患者对于健康节目的关注度更高（*P* < 0.001），并且对于医务人员整体印象更好（*P*=0.001）。

**1:** 肿瘤患者社会人口学信息概况 Sociodemographic characteristics of cancer patients

Variable	Total (*n*=619)			Attended			Not attended		*χ*^2^/*t*	*P*
	*N*	%		*N*	%		*N*	%		
Age, years (Mean±SD)	53.4±12.7			53.6 ±13.4			53.6±13.4		0.62	0.538
Gender									2.44	0.118
Male	307	49.6%		128	53.6%		179	47.1%		
Marriage									0.00	0.964
Single/divorced/widowed	47	7.6%		18	7.5%		29	7.6%		
Married	572	92.4%		221	92.5%		351	92.4%		
Education^a^									1.84	0.399
Junior high and below	227	36.8%		86	36.0%		141	37.3%		
Senior high	268	43.4%		111	46.4%		157	41.5%		
College graduate or higher	122	19.8%		42	17.6%		80	21.2%		
Occupation^b^									7.63	0.054
Full-time employees	204	34.3%		68	29.3%		136	37.5%		
Peasantry	108	18.2%		45	19.4%		63	17.4%		
Unemployed	137	23.0%		50	21.6%		87	24.0%		
Retired	146	24.5%		69	29.7%		77	21.2%		
Relatives or friends in medical industry									0.02	0.892
Yes	217	35.1%		83	34.7%		134	35.3%		
Religion^c^									2.57	0.277
Have religious belief	64	10.4%		21	8.8%		43	11.3%		
Watch health TV show									22.57	< 0.001
Often	164	26.5%		60	25.1%		104	27.4%		
Sometimes	190	30.7%		97	40.6%		93	24.5%		
Rarely	189	30.5%		65	27.2%		124	32.6%		
Never	76	12.3%		17	7.1%		59	15.5%		
Impression of medical staff									11.07	0.001
Good	363	58.6%		160	66.9%		203	53.4%		
General/not bad/bad	256	41.4%		79	33.1%		177	46.6%		
^a^n=617; ^b^n=595; ^c^n=618										

在肿瘤患病情况上（表 2），相较未参加过临床试验的患者，参加过临床试验的患者有以下几个特点。患病时间更长，中位时长为1, 032 d（*P* < 0.001），病种更集中在肺、乳腺、消化相关肿瘤（*P*=0.007），临床分期更晚，中晚期患者占45.6%（*P*=0.008），88.7%的患者接受过内科治疗（*P* < 0.001），并且自评治疗效果更好（*P*=0.001）。

**2:** 肿瘤患者患病治疗信息概况 Cancer status and treatment of cancer patients

Variables	Total (*n*=594)			Attended			Not attended			*χ*^2^	*P*
	*N*	%		*N*	%		*N*	%			
Days of diagnosed [P50(P25-P75)]	500 (118-1331)			1, 032 (573-1998)			198 (76-765)			11.40	< 0.001
Cancer type										19.50	0.007
Lung	157	25.4%		65	27.2%		92	24.2%			
Breast	133	21.5%		61	25.5%		72	18.9%			
Digestive system	155	25.0%		64	26.8%		91	23.9%			
Genital system	51	8.2%		8	3.3%		43	11.3%			
Lymphatic system	35	5.7%		12	5.0%		23	6.1%			
Head and Neck	20	3.2%		6	2.5%		14	3.7%			
Urinary system	26	4.2%		12	5.0%		14	3.7%			
Others	42	6.8%		11	4.6%		31	8.2%			
Stage										7.13	0.008
Early	118	19.1%		44	18.4%		74	19.5%			
Advanced	207	33.4%		109	45.6%		98	25.8%			
Not clear	294	47.5%		86	36.0%		208	54.7%			
Previous treatment										52.34	< 0.001
Medical treatment	448	72.4%		212	88.7%		236	62.1%			
Other treatment	121	19.5%		21	8.8%		100	26.3%			
None	50	8.1%		6	2.5%		44	11.6%			
Treatment phase										21.23	< 0.001
Not start treating	68	11.4%		9	3.9%		59	16.2%			
On-therapy	411	69.2%		175	76.4%		236	64.7%			
reexamination	115	19.4%		45	19.7%		70	19.2%			
Self-assessed therapeutic effect										11.64	0.001
Great/Good	351	56.7%		156	65.3%		195	51.3%			
General/Bad/Not known	268	43.3%		83	34.7%		185	48.7%			
Other treatments include surgery, radiation and traditional medicine.											

### 2.2 临床试验的认知情况

患者对于临床试验认知的具体情况见表 3和图 1。338例（54.6%）患者对临床试验整体认知较为正确，但仍有44例（7.1%）患者认为“参与临床试验患者是科学研究的牺牲品”，参加试验并没有显著提升患者对于临床试验整体认知的正确性（57.7% *vs* 52.6%, *P*=0.214）。其中，在以下6个临床试验认知维度的评价中，参加过试验的患者的认知程度较未参加过试验的患者均有一定的提升，包括研究意义（86.2% *vs* 77.6%, *P*=0.008）、风险收益告知（91.2% *vs* 71.6%, *P* < 0.001）、资料保密（73.2% *vs* 59.7%, *P*=0.001）、自愿参与（95.8% *vs* 76.3%, *P* < 0.001）、随时退出（86.6% *vs* 68.2%, *P* < 0.001）、费用影响（62.8% *vs* 39.2%, *P* < 0.001）。在费用补偿（51.5% *vs* 48.7%, *P*=0.501）和试验相关法律法规保护（52.3% *vs* 45.5%, *P*=0.101）上，参加过临床试验的患者认知度并没有得到显著的提高。

**3:** 参加过和未参加过临床试验的肿瘤患者临床试验认知情况 Comparison of awareness of patients who attended clinical trials before and those who did not

Variables	Total			Attended			Not attended	
	*N*	%		*N*	%		*N*	%
Overall understanding of attending clinical trials								
Better treatment with lower risk	237	38.3%		95	39.7%		142	37.4%
Victim of scientific research	44	7.1%		6	2.5%		38	10.0%
Free treatment with both risk and benefit	338	54.6%		138	57.7%		200	52.6%
Clinical trials are meaningful for medical development and patient treatment.									
Yes	501	80.9%		206	86.2%		295	77.6%
Unsure	50	8.1%		18	7.5%		32	8.4%
No	6	1.0%		0	0.0%		6	1.6%
Not understand	62	10.0%		15	6.3%		47	12.4%
Doctors will inform risk of clinical trials before attending.								
Yes	490	79.2%		218	91.2%		272	71.6%
Unsure	39	6.3%		7	2.9%		32	8.4%
No	12	1.9%		4	1.7%		8	2.1%
Not understand	78	12.6%		10	4.2%		68	17.9%
Doctors will keep the information of cases confidential during the clinical trials.								
Yes	402	64.9%		175	73.2%		227	59.7%
Unsure	48	7.8%		14	5.9%		34	9.0%
No	33	5.3%		16	6.7%		17	4.5%
Not understand	136	22.0%		34	14.2%		102	26.8%
Participants are voluntary to participate clinical trials.								
Yes	519	83.8%		229	95.8%		290	76.3%
Unsure	36	5.8%		5	2.1%		31	8.2%
No	6	1.0%		1	0.4%		5	1.3%
Not understand	58	9.4%		4	1.7%		54	14.2%
Participants are free to withdraw at any time during clinical trials.								
Yes	466	75.3%		207	86.6%		259	68.2%
Unsure	30	4.8%		7	2.9%		23	6.1%
No	36	5.8%		10	4.2%		26	6.8%
Not understand	87	14.1%		15	6.3%		72	18.9%
The change of medical expenses after attending clinical trials								
Increase	38	6.1%		18	7.5%		20	5.3%
Unsure	75	12.1%		30	12.6%		45	11.8%
Decrease	299	48.3%		150	62.8%		149	39.2%
Not understand	207	33.4%		41	17.1%		166	43.7%
Participants will get compensation of damage as a result of clinical trials.								
Yes	308	49.8%		123	51.5%		185	48.7%
Unsure	74	12.0%		38	15.9%		36	9.5%
No	36	5.8%		14	5.9%		22	5.8%
Not understand	201	32.5%		64	26.8%		137	36.1%
There are related laws of scientific and ethical issues in clinical trials.								
Yes	298	48.1%		125	52.3%		173	45.5%
Unsure	26	4.2%		13	5.4%		13	3.4%
No	28	4.5%		11	4.6%		17	4.5%
Not understand	267	43.2%		90	37.7%		177	46.6%

**1:**
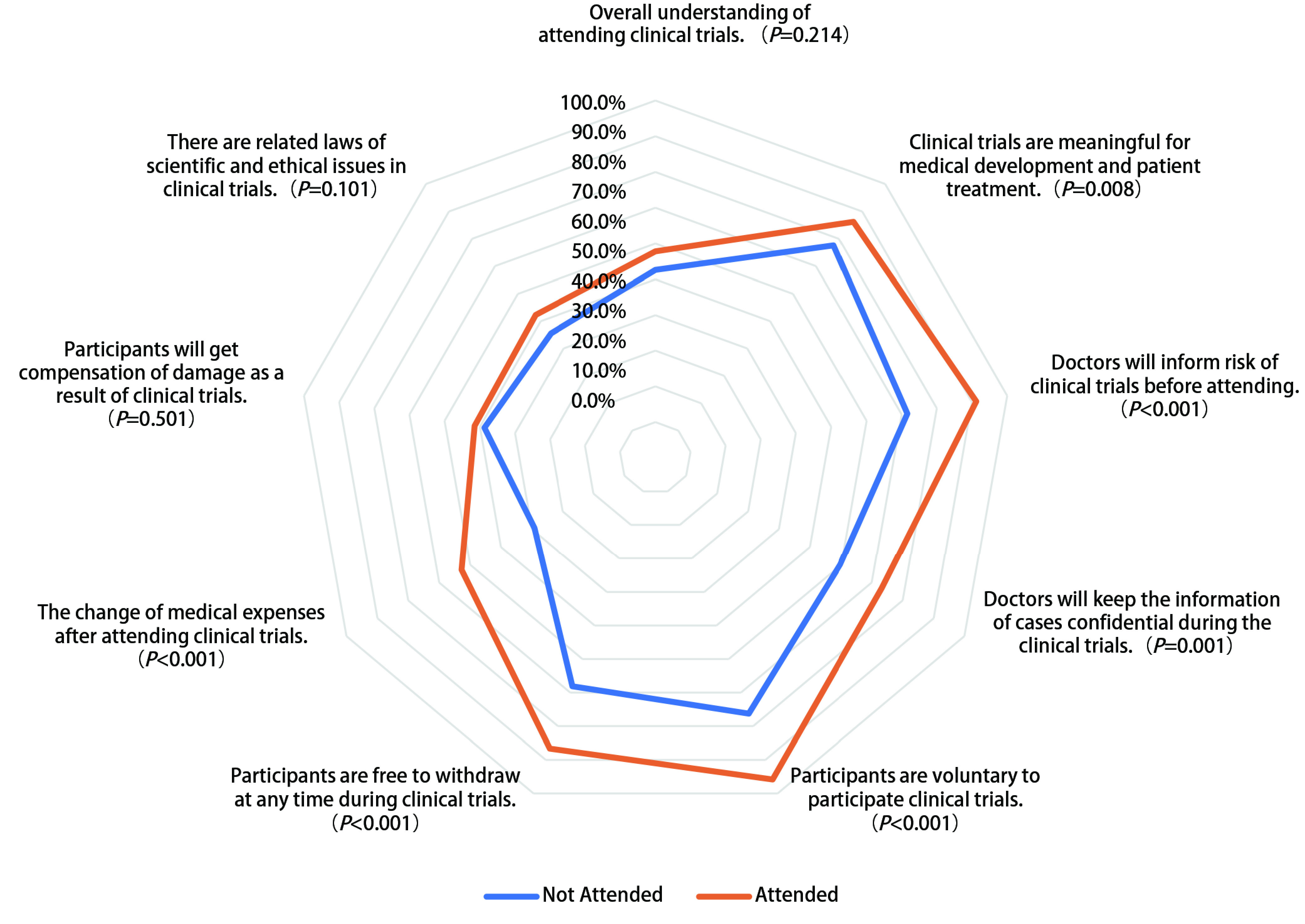
参加过和未参加过临床试验的肿瘤患者临床试验认知情况 Comparison of awareness of patients who attended clinical trials before and those who did not

### 2.3 认知度的单因素影响分析

认知度的单因素分析显示婚姻状况（*P*=0.002）、对医务人员印象（*P*=0.0.017）、确诊时间（*P* < 0.001）、既往治疗史（*P*=0.002）和临床试验参与情况（*P* < 0.001）与患者认知度的水平显著相关（表 4），其他因素如年龄、性别、教育程度、职业、医疗行业亲友、宗教信仰、对健康节目的关注程度、肿瘤分期、治疗阶段以及自评疗效均未发现与对临床试验的认知水平相关（*P* > 0.05）。

**4:** 肿瘤患者临床认知度的单因素影响分析 Univariate analysis of variables associated with clinical trial awareness

Variables	Lower cognition group			Higher cognition group		*χ*^2^	*P*
	*N*	%		*N*	%		
Age (yr)						0.16	0.925
≤50	136	59.6%		92	40.4%		
50-60	100	58.5%		71	41.5%		
≥60	133	60.5%		87	39.5%		
Gender							0.744
Male	185	60.3%		122	39.7%	0.11	
Female	184	59.0%		128	41.0%		
Marriage						9.60	0.002
Single/divorced/widowed	18	38.3%		29	61.7%		
Married	351	61.4%		221	38.6%		
Education^b^						5.89	0.053
Junior high or below	149	65.6%		78	34.4%		
Senior high school	152	56.7%		116	43.3%		
College graduate or higher	66	54.1%		56	45.9%		
Occupation^c^						6.40	0.094
Full-time employees	111	54.4%		93	45.6%		
Peasantry	74	68.5%		34	31.5%		
Unemployed	84	61.3%		53	38.7%		
Retired	83	56.8%		63	43.2%		
Relatives or friends in medical industry						2.58	0.108
Yes	120	55.3%		97	44.7%		
No	249	61.9%		153	38.1%		
Religion^a^						0.28	0.526
Have religious belief	41	64.1%		23	35.9%		
Not have religious belief	327	59.0%		227	41.0%		
Watch health TV show						4.27	0.234
Often	100	61.0%		64	39.0%		
Sometimes	102	88.0%		88	46.3%		
Rarely	118	62.4%		71	37.6%		
Never	49	64.5%		27	35.5%		
Impression of medical staff						5.73	0.017
Great	202	55.6%		161	44.4%		
General/not bad/bad	167	65.2%		89	34.8%		
Time of diagnosed, years						15.48	< 0.001
< 1	195	67.9%		92	32.1%		
1-2	47	53.4%		41	46.6%		
> 2	127	52.0%		117	48.0%		
Clinical stage						3.58	0.058
Early	71	60.2%		47	39.8%		
Advanced	102	49.3%		105	50.7%		
Previous treatment						12.21	0.002
Medical treatment	248	55.4%		200	44.6%		
Other treatment	86	71.1%		35	28.9%		
None	35	70.0%		15	30.0%		
Treatment phase						1.80	0.407
Not start treating	44	64.7%		68	35.3%		
On-therapy	237	27.7%		411	42.3%		
reexamination	72	62.6%		115	37.4%		
Self-assessed therapeutic effect						0.49	0.483
Great/Good	205	58.4%		146	41.6%		
General/Bad/Not known	164	61.2%		104	38.8%		
Previous attended clinical trials						28.04	< 0.001
Yes	122	32.1%		258	67.9%		
No	128	53.6%		111	46.4%		
^a^n=618; ^b^n=617; ^c^n=595								

### 2.4 认知度的多因素影响分析

多因素分析显示，是否参加过临床试验、对医务人员的印象、婚姻状况及职业是认知度的独立影响因素（表 5）。结果显示，参加过试验（OR=1.83, 95%CI: 1.11-3.00）、未婚/离异（OR=5.04, 95%CI: 1.73-14.66）、退休（OR=2.53, 95%CI: 1.16-5.50）患者认知度较高，对医务人员印象较好/一般/差（OR=0.43, 95%CI: 0.26-0.72）。

**5:** 肿瘤患者临床试验认知度的多因素分析 Multivariate analysis of variables associated with clinical trial awareness

Variables	*β* value	SE	Wald *X*^2^	*P*	OR (95%CI)
Previous attended clinical trials					
No	-	-	-	-	1.00
Yes	0.60	0.25	0.02	0.018	1.83 (1.11-3.00)
Marriage					
Married	-	-	-	-	1.00
Single/divorced/widowed	1.62	0.54	8.83	0.003	5.04 (1.73-14.66)
Occupation					
Peasantry	-	-	-	-	1.00
Retired	0.93	0.40	5.45	0.020	2.53 (1.16-5.50)
Full-time employees	0.64	0.39	2.73	0.099	1.90 (0.89-4.09)
Unemployed	0.34	0.41	0.66	0.418	1.40 (0.62-3.15)
Impression with doctors					
Good	-	-	-	-	1.00
General/not bad/bad	-0.84	0.26	10.44	0.001	0.431 (0.259-0.718)

## 3 讨论

基于中国医学科学院肿瘤医院617例患者的调查研究显示，肿瘤患者对临床试验的认知度较为有限，仅有54.6%肿瘤患者对临床试验整体看法正确。参加试验能够在一定程度上提高患者对于临床试验的认知，但限于部分认知维度。除外临床试验参与情况，多因素分析还提示，对医务人员印象好、未婚/离异以及退休的患者认知程度相对较高。

与我国既往认知度研究相比（31.02%），本研究纳入患者对于临床试验的认知程度有所提高长^[11]^；但相较于既往国外认知度调查结果（60%-80%）仍有较大差距^[6, 13-15]^。主要是有以下几点原因：（1）对认知评价方式的差异，大多数国际研究在衡量认知度时是在评估患者或民众是否“听过”临床试验，很少评估被调查者是否对试验有着正确的认识^[6]^。少数研究发现在知晓率很高的情况下，正确理解临床试验含义的人占比很少^[16]^。（2）国内缺乏对于临床试验的宣传和教育。网络的使用率上升和宣传教育活动与临床试验认知度的提高息息相关^[8-10]^，但我国缺少临床试验研究有关的权威招募平台和宣传，患者缺乏了解临床试验的有效渠道。

国内患者认知度与其对医护人员印象十分相关，对医护人员印象越好的患者往往认知度就越高。其中一个原因是我国医疗信息不对称，患者缺乏临床试验信息获取渠道进而极大依赖于医护人员以提高认知。一方面提示，促进医患关系、让医务人员广泛的参与到患者教育中对于提高患者临床试验认知度的重要作用；另一方面，这也与本研究团队同期研究^[4]^及既往两项研究发现一致^[13, 17]^，主治医生对患者临床试验参与决策具有关键作用。

对比9个维度认知正确人群比例，肿瘤患者在自愿参与、研究意义、受益风险告知、随时退出和资料保密维度认知相对较好。在临床试验法律法规保护、医疗费用影响及经济补偿方面的认知最差，低于50%。此外，本研究首次对比未参加过试验和参加过试验患者的认知度差异，发现参加过试验患者认知度有一定的提高。参加过试验患者在自愿参加、随时退出和资料保密等维度与既往*meta*研究结果较为一致^[18]^。但研究也发现，参加过试验患者存在认知提高不明显以及部分维度（经济补偿、法律保护以及临床试验整体看法）无提高等问题。提示在临床试验知情同意过程中，存在知情告知不充分或者告知有效性不足的问题。因此，在实际临床试验管理过程中，伦理审核需重点把关知情同意书设计完整性以及可读性，同时医院需有针对性的提高研究医生对知情同意过程重要性的认识，保证知情讨论时间充足和效果充分^[19]^。

本研究创新性地探索了肿瘤患者临床试验认知度的影响因素，并进行了参加过试验和未参加过试验患者在临床试验认知方面的差异比较，为中国医学科学院肿瘤医院乃至我国提升肿瘤患者临床试验认知的相关策略制定、促进临床试验管理工作优化提供了科学依据。但本研究存在一些局限性。首先，本次调查是单中心调查，人群代表性和结果外推性不足。其次，本研究存在人群选择偏倚，受实际工作开展限制，采取了方便抽样，而非随机抽样。另外，认知度的指标定义和测量方法尚无共识，尽管本研究的问卷设计和指标测量经过国家癌症中心专家认定，但也存在与其他研究可比性不充分的问题。

总体来说，我国肿瘤患者目前对临床试验认知度较为有限，尤其是在经济补偿、费用影响以及法律保护维度。促进医患和谐、建立有效临床试验信息公示平台以及开展临床试验知识普及对提高患者认知度十分有必要。同时，针对性地加强临床试验知情同意告知的充分性和有效性也是未来工作的重要方向。

## Author contributions

Huang HY, Fang Y, Shi JF, Li N conceived and designed the study. Huang HY, Fang Y, Fang H, Wu DW, Wang SH, Bai Y, Yu AQ, Wang H, Sun C, Yu Y, Fan Y, Yang C, Shi JF, Li N performed the experiments. Huang HY, Fang Y, Shi JF, Li N analyzed the data. Huang HY, Yang C, Shi JF, Shi JF contributed analysis tools. Huang HY, Fang Y, Shi JF, Li N provided critical inputs on design, analysis, and interpretation of the study. All the authors had access to the data. All authors read and approved the final manuscript as submitted.
